# Clinical Plausibility in Large Language Model Robustness Testing for Medicine: A Scoping Review

**DOI:** 10.1007/s10916-026-02405-1

**Published:** 2026-05-11

**Authors:** Yu Chang, Ming-Hong Hsieh, Po-Chung Ju, Yi-Chun Liu, Cheng-Chen Chang

**Affiliations:** 1 Department of Psychiatry, Chung Shan Medical University Hospital, Taichung, 40106 Taiwan; 2 School of Medicine, Chung Shan Medical University, No. 110, Jianguo N. Rd., Sec. 1, Taichung City, 40106 Taiwan; 3 Post Baccalaureate Medicine, National Chung Hsing University, Taichung, Taiwan; 4 Present Address: Department of Psychiatry, Changhua Christian Hospital, No.135, Nanhsiao Street, Changhua, 500 Taiwan; 5 Department of Psychiatry, Changhua Christian Children’s Hospital, Changhua, Taiwan; 6 Department of Health Business Administration, Hungkuang University, Taichung City, Taiwan

**Keywords:** Large language model, Clinical decision support system, Robustness testing, Clinical plausibility, Red teaming

## Abstract

Large language models (LLMs) show promise in medical applications, yet their translation into clinical practice requires rigorous validation. Current robustness testing often employs adversarial approaches borrowed from AI safety, raising questions about their alignment with authentic clinical scenarios. To systematically map methodologies used for robustness testing of LLMs in medical contexts and assess their clinical plausibility. A scoping review was conducted following PRISMA-ScR guidelines, searching PubMed, Embase, Web of Science, IEEE Xplore, ACM Digital Library, arXiv, and MedRxiv from January 2023 to September 2025. Two independent physician reviewers screened 5,331 articles, extracting data on testing methodologies, medical domains, expert involvement, and clinical plausibility. Thirty-three studies met inclusion criteria, predominantly from 2025 (82%). The most common robustness testing approaches were misleading prompts (49%) and adversarial prompts (39%). Only 33% of studies designed tests clearly mimicking plausible clinical scenarios. While 58% reported expert involvement, the depth of integration varied considerably. Studies predominantly addressed mixed medical domains (73%) rather than specialized fields. The emerging literature suggests that LLM robustness testing in medicine often emphasizes technical vulnerability detection, with fewer studies examining clinically plausible scenarios of routine use. Future frameworks should complement adversarial testing with clinically grounded, longitudinal, and specialty focused evaluations to support deployment-relevant inference.

**Supplementary Information:**

The online version contains supplementary material available at 10.1007/s10916-026-02405-1.

## Introduction

Large language models (LLMs) are rapidly being explored for diverse medical applications [1]. However, translating this technology into clinical practice is a significant challenge due to the field’s low risk tolerance and high professional demands [2–4]. Building trust in LLMs requires extensive validation [5], which is particularly important given the inherent uncertainties of medicine [6].

While LLMs demonstrate impressive knowledge on standardized benchmarks [7, 8], reasoning-oriented models, for example DeepSeek-R1 [9], increasingly perform well on structured clinical vignettes that align with elements of clinician reasoning. Nonetheless, biases and variable reliability under real-world inputs may constrain translation to complex systems such as clinical decision support systems (CDSS). A critical barrier to wider adoption is the need to ensure their robustness [10]. For the purpose of this review, reliability refers to the consistency and dependability of model outputs, whereas robustness refers to the ability to maintain performance despite perturbations, distributional shifts, or changes in context [11].

However, robustness evaluations can diverge from clinical reality in two ways. Evaluation datasets are not always aligned with routine clinical data in structure, completeness, and workflow context, which can introduce dataset shift and limit generalizability [12, 13]. In parallel, many robustness evaluations draw on AI safety and software engineering approaches, including adversarial attacks and red teaming, to elicit model failures [14, 15]. Misleading clinical inputs, such as incomplete or contradictory information, differ from adversarial inputs that intentionally exploit model vulnerabilities. This distinction raises an important question: to what extent do current robustness tests capture the uncertainty and context dependence of routine clinical practice?

The integration of LLMs into medicine is an interdisciplinary field [16], and a disconnect can exist between LLM and medical experts [17]. This can impact the design of robustness tests, which must account for the profound influence of clinical uncertainty and subjectivity on model outputs. Therefore, a critical step is to ensure that testing methodologies are aligned with clinical reality. Only through appropriately aligned evaluations can we truly understand the practical utility of these models.

To address this gap, we conducted a scoping review to map the existing literature on robustness testing for LLMs in medical contexts. This review systematically examines the methodologies employed and assesses their alignment with plausible clinical scenarios. By identifying the limitations of current approaches, we aim to guide future research toward developing more clinically meaningful evaluations, thereby enhancing the substantive impact and fostering the responsible integration of LLMs into healthcare.

## Method

### Protocol and Registration

This scoping review was preregistered with OSF Registries and was designed in accordance with its publicly available protocol (DOI: 10.17605/OSF.IO/CHYVA). Deviation from the registered protocol is provided in the supplementary Method S1. The methodology and reporting strictly adhere to the Preferred Reporting Items for Systematic Reviews and Meta-Analyses extension for Scoping Reviews (PRISMA-ScR), and the completed checklist is provided in the supplementary Method S2.

### Eligibility Criteria

This review considered studies that evaluated the robustness of LLMs within a medical context. The search period was defined from January 1, 2023, to September 23, 2025. The start date was chosen to align with the emergence of highly capable and widely researched LLM systems [7]. All publication types, including original research articles and research letters, were eligible for inclusion, provided they described both the methodology and the results of a robustness test. Conversely, studies were excluded if they only presented a protocol or a conceptual framework without empirical results. Further exclusion criteria were applied to remove studies that lacked a discernible comparative benchmark, although studies using established historical benchmarks in place of a direct control group were retained. We excluded studies where the tested application was used exclusively for information summarization without an explicit inferential or decision-support component. For example, tasks such as generating patient discharge summaries or summarizing physician notes for billing purposes were deemed outside the scope of this review. No language restrictions were imposed during the selection process.

### Information Sources and Search Strategy

A comprehensive literature search was executed on September 23, 2025. Publication status and source type were recorded as of this search date. The search strategy was designed to be interdisciplinary, encompassing major medical and technical databases, including PubMed, Embase, Web of Science (Core Collection), IEEE Xplore, and the ACM Digital Library. The search query was structured around the union of three core concepts: large language models, medical applications, and robustness. For example, the PubMed search string was: ((“large language model“[tiab] OR LLM[tiab] OR “generative pre-trained transformer“[tiab] OR GPT[tiab] OR ChatGPT[tiab] OR Gemini[tiab] OR Claude[tiab]) AND (medical[tiab] OR clinical[tiab] OR healthcare[tiab] OR “patient care“[tiab] OR diagnosis[tiab] OR treatment[tiab]) AND (reliability[tiab] OR robustness[tiab] OR “adversarial*“[tiab] OR “prompt*“[tiab] OR “red team*“[tiab] OR “stress test*“[tiab] OR “uncertainty“[tiab])). The detailed search strategies for each database are provided in the supplementary Method S3. To account for the rapid pace of innovation in this field, the search was extended to the preprint servers arXiv and MedRxiv. Due to platform limitations, these servers were queried programmatically using a custom R script (supplementary Method S4) that interacted with their respective application programming interfaces (APIs).

### Selection and Data Charting Process

The study selection and data charting process was conducted by two independent, board-certified physicians (YC, YCL). Initial screening was facilitated by the Rayyan platform, where duplicates were removed first automatically by matching DOIs and titles with a similarity of 97% or higher, followed by a manual deduplication step. In cases where a study was available as both a preprint and a peer-reviewed publication, the latter was retained. The reviewers then proceeded with a two-stage screening process, first evaluating titles and abstracts, and then conducting a full-text review of potentially relevant articles. Throughout this process, Rayyan’s AI-driven recommendation features were used to assist in prioritizing articles, but all final inclusion and exclusion decisions were made manually. Any disagreements at either stage were resolved through discussion and consensus; if a consensus could not be reached, a third senior specialist (CCC) was consulted to make the final determination.

Following selection, the same two reviewers independently charted data from all included studies using a standardized extraction procedure. For studies comprising multiple sub-experiments, data extraction was limited to components directly relevant to clinical decision-making. As a single study could employ multiple methodological approaches, each study could be coded under several categories. This procedure was designed to capture methodological attributes without requiring deep subspecialty knowledge. Specifically, the extracted data items included: basic bibliographic information (author, title, journal, year, country); the nature of the task (e.g., multiple-choice questions, classification, open-ended generation); the medical domain (e.g., a specific specialty or mixed domains); the specific method used for robustness testing, classified under pre-specified categories (e.g., “Misleading prompts,” which introduce confusing, incomplete, or contradictory information within a clinically plausible scenario to assess robustness under clinical uncertainty; “Adversarial prompts,” which are intentionally designed to exploit model vulnerabilities or induce unstable, unsafe, or unintended outputs rather than to simulate routine clinical uncertainty; “Context-variation prompts” that vary the clinical context); and the nature of expert involvement, capturing if an expert was present (Yes/No), their professional field, and their reported role (e.g., study design, prompt or case design, output assessment, or interpretation), when available.

Finally, a key variable was charted to determine if the test was designed to mimic a real-world clinical scenario (Yes/No/Mixed). This three-level categorical classification was intended as a pragmatic study-level mapping variable for scoping purposes, rather than a comprehensive measure of multidimensional clinical realism. We defined Yes as a question or task that a practicing clinician could plausibly pose within routine clinical communication or documentation to support decision-making, focusing on the plausibility of interaction intent and representation rather than information correctness, since real clinical inputs may be incomplete or contain misinformation. Studies were coded as No when their primary evaluation relied on adversarial intent, nonclinical input representations, or interaction patterns unlikely to occur in routine clinical workflows. For studies coded as No, we conducted an analysis of the reasons for implausibility, developing a taxonomy of violation types through the same two reviewers’ discussion and consensus.

### Synthesis of Results

The primary objective of this review is to map the existing methodologies for robustness testing rather than to synthesize study outcomes. Given the anticipated heterogeneity in the models, datasets, and testing protocols employed, a quantitative synthesis or formal quality appraisal of the study results was deemed inappropriate and was not performed. Instead, the extracted data will be synthesized narratively. Descriptive statistics will be used to summarize the frequencies of different study characteristics, testing methods, and the extent of clinical realism. We will then perform a thematic analysis to categorize the identified techniques, critically examining these categories to identify which aspects of clinical uncertainty are being addressed and to highlight significant gaps where current testing paradigms may not yet align with authentic clinical practice.

## Results

**Fig. 1:**
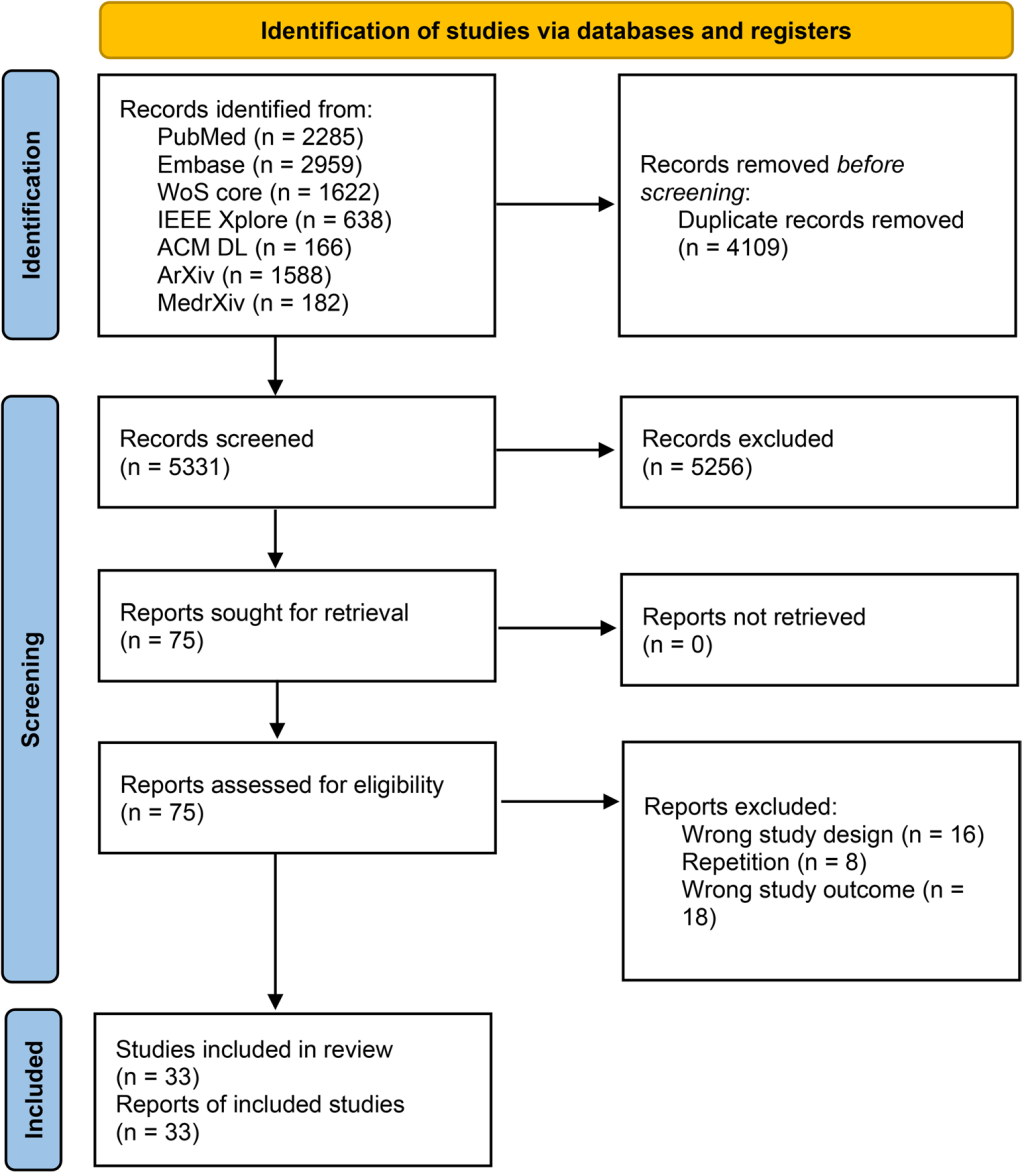
PRISMA-ScR (scoping review) flow diagram

The initial search yielded 9,440 articles, which was reduced to 5,331 after the removal of duplicates. Following title and abstract screening, 75 articles were selected for full-text review, from which a final set of 33 studies met the inclusion criteria. A detailed overview of this selection process is presented in the PRISMA flow diagram (Fig. 1). Details of the excluded full-text articles and the corresponding reasons for exclusion are presented in the supplementary Result S1.

A descriptive analysis of the included studies reveals several key trends **(**Table 1**)**. The entire body of literature emerged recently, with no studies published in 2023, six (18%) in 2024, and a significant acceleration to 27 studies (82%) in the first three quarters of 2025. Geographically, research from the United States was most prominent, accounting for 15 (45%) of the included publications. In terms of publication venue, a majority of the studies, 17 in total (52%), were posted on the arXiv pre-print server.

**Table 1:** Overview of study characteristics and methodologies

Study ID	Title	Journal/Source at search	Year	Country	Task	Domain	What method	Expert Matched (Y/*N*)	Domain of expertise	Role of expert involvement	Mimic real-world(Y/*N*/Mixed)	Reason
[35] Ali (2025)	AtlasGPT: a language model grounded in neurosurgery with domain-specific data and document retrieval	Journal of Neurosurgery	2025	US	Open question	Neurosurgery	Adversarial prompt	Y	Neurosurgery	Design	N	Intentional Misrepresentation
[36] Zada (2025)	Medical Misinformation in AI-Assisted Self-Diagnosis: Development of a Method (EvalPrompt) for Analyzing Large Language Models	JMIR Formative Research	2025	Canada	Open question	Mixed medical	Ablation	Y	Mixed	Design and assessment	N	Workflow Violation
[37] Clusmann (2025)	Prompt injection attacks on vision language models in oncology	Nature Communications	2025	Germany	Open question	Radiology	Adversarial prompt	Y	Radiology	Assessment	N	Intentional Misrepresentation
[38] Omar (2025)	Multi-model assurance analysis showing large language models are highly vulnerable to adversarial hallucination attacks during clinical decision support	Communications Medicine	2025	US	Open question	Mixed medical	Adversarial prompt	Y	Mixed	Design and assessment	N	Intentional Misrepresentation
[23] Shoval (2025)	A controlled trial examining large Language model conformity in psychiatric assessment using the Asch paradigm	BMC Psychiatry	2025	Israel	MCQ	Mixed medical	Misleading prompt	N	Not involved	Not involved	Y	
[24] Dergaa (2024)	ChatGPT is not ready yet for use in providing mental health assessment and interventions	Front. Psychiatry	2024	Tunisia	Open question	Psychiatry	Context-variation prompt	Y	Psychiatry	Design and assessment	Y	
[39] Xiong (2025)	Evaluating the Performance of Large Language Models (LLMs) in Answering and Analysing the Chinese Dental Licensing Examination	European Journal of Dental Education	2025	China	MCQ	Dental	Adversarial prompt	Y	Dentist	Design	N	Intentional Misrepresentation
[40] Liu (2025)	Assessing the Quality of Artificial Intelligence Responses and Resistance to Sycophancy in Providing Patient-centered Medical Advice on Gastroesophageal Reflux Disease	Clinical Gastroenterology and Hepatology	2025	US	Open question	Gastroenterology	Misleading prompt	N	Not gastroenterology	Design and assess	Y	
[41] Safrai (2024)	Does small talk with a medical provider affect ChatGPT’s medical counsel? Performance of ChatGPT on USMLE with and without distractions	PLOS ONE	2024	Israel	MCQ and Open question	Mixed medical	Misleading prompt	N	Obstetrics and Gynecology, not matched	Assessment	N	Intentional Misrepresentation
[42] Campo (2025)	Analyses of different prescriptions for health using artificial intelligence: a critical approach based on the international guidelines of health institutions	Health Information Science and Systems	2025	Brazil	Open question	Mixed medical	Context-variation prompt	Y	Mixed	Design and assessment	N	Workflow Violation
[43] Schmidgall (2024)	Evaluation and mitigation of cognitive biases in medical language models	npj Digital Medicine	2024	US	MCQ	Mixed medical	Misleading prompt	N	Only Geriatric Medicine and Gerontology	Design	N	Intentional Misrepresentation
[22] Chang (2025)	Evaluating the Impact of Authoritative and Subjective Cues on Large Language Model Reliability for Clinical Inquiries: An Experimental Study	medRxiv	2025	TW	Classification	Psychiatry	Misleading prompt	Y	Psychiatry	Design	Y	
[44] Lee (2025)	Manipulating medical advice through stealth prompt injection in large language models: An experimental study on vulnerabilities and patient safety risks	SSRN	2025	Korea	Open question	Mixed medical	Adversarial prompt	Y	Mixed	Design and assessment	N	Intentional Misrepresentation
[45] Chen (2025)	When Helpfulness Backfires: LLMs and the Risk of Misinformation Due to Sycophantic Behavior	Research Square	2025	US	Open question	Mixed medical	Misleading prompt	Y	Mixed	Design and assessment	N	Intentional Misrepresentation
[46] Yang (2024)	Adversarial Attacks on Large Language Models in Medicine	arXiv	2024	US	Open question	Mixed medical	Adversarial prompt	N	Not involved	Not involved	N	Intentional Misrepresentation
[47] Huang (2025)	Medical MLLM is Vulnerable: Cross-Modality Jailbreak and Mismatched Attacks on Medical Multimodal Large Language Models	Proceedings of the AAAI Conference on Artificial Intelligence	2025	China	Open question	Radiology	Adversarial prompt	N	Not involved	Not involved	N	Intentional Misrepresentation
[48] Ness (2024)	MEDFUZZ: Exploring the Robustness of Large Language Models in Medical Question Answering	arXiv	2024	US	Open question	Mixed medical	Adversarial prompt	N	Not involved	Not involved	N	Intentional Misrepresentation
[49] Yang (2024)b	Ensuring Safety and Trust: Analyzing the Risks of Large Language Models in Medicine	arXiv	2024	US	MCQ	Mixed medical	Misleading prompt and Adversarial prompt	N	Mixed, not totally matched	Design	Mixed	Intentional Misrepresentation
[50] Kim (2025)	Limitations of Large Language Models in Clinical Problem-Solving Arising from Inflexible Reasoning	arXiv	2025	Canada	MCQ	Mixed medical	Context-variation prompt	Y	Mixed, most neurologist	Design	Y	
[21] Lim (2025)	Susceptibility of Large Language Models to User-Driven Factors in Medical Queries	arXiv	2025	Korea	MCQ	Mixed medical	Misleading prompt	Y	Mixed	Design	Y	
[51] Subedi (2025)	The Reliability of LLMs for Medical Diagnosis: An Examination of Consistency, Manipulation, and Contextual Awareness	Journal of Advanced Artificial Intelligence, Engineering and Technology	2025	Not known	Blank filling	Mixed medical	Misleading prompt	Y	Not known	Design	Mixed	Intentional Misrepresentation
[52] Zhu (2025)	Cancer-Myth: Evaluating Large Language Models on Patient Questions with False Presuppositions	arXiv	2025	US	Open question	Mixed medical	Misleading prompt	Y	Not known	Design	Y	
[53] Vishwanath (2025)	Medical large language models are easily distracted	arXiv	2025	US	MCQ	Mixed medical	Misleading prompt	Y	Mixed	Design	N	Intentional Misrepresentation
[54] Chen (2025)b	MedSentry: Understanding and Mitigating Safety Risks in Medical LLM Multi-Agent Systems	arXiv	2025	China	Classification	Mixed medical	Misleading prompt and Adversarial prompt	Y	Not known	Design	Mixed	Intentional Misrepresentation
[55] Chen (2025)c	CARES: Comprehensive Evaluation of Safety and Adversarial Robustness in Medical LLMs	arXiv	2025	US	Open question	Mixed medical	Adversarial prompt	N	Not involved	Not involved	N	Intentional Misrepresentation
[56] Balazadeh (2025)	Red Teaming Large Language Models for Healthcare	arXiv	2025	Canada	Open question	Mixed medical	Misleading prompt and Adversarial prompt	Y	Not known	Design and assessment	Y	
[57] Sadanandan (2025)	VSF-Med: A Vulnerability Scoring Framework for Medical Vision-Language Models	arXiv	2025	US	Image interpretation	Radiology	Adversarial prompt and input	N	Not involved	Not involved	N	Intentional Misrepresentation
[58] Gourabathina (2025)	The MedPerturb Dataset: What Non-Content Perturbations Reveal About Human and Clinical LLM Decision Making	arXiv	2025	US	Classification	Mixed medical	Context-variation prompt	N	Medical student	Assessment	N	Attribute Mutation
[59] Li (2025)	COUNSELBENCH: A Large-Scale Expert Evaluation and Adversarial Benchmarking of Large Language Models in Mental Health Question Answering	arXiv	2025	US	Open question	Mixed medical	Misleading prompt	Y	Mental mixed	Design and assessment	Y	
[60] Pan (2025)	Beyond Benchmarks: Dynamic, Automatic And Systematic Red-Teaming Agents For Trustworthy Medical Language Models	arXiv	2025	US	MCQ	Mixed medical	Misleading prompt	N	Not involved	Not involved	N	Intentional Misrepresentation
[61] Vijayaraj (2025)	Embeddings to Diagnosis: Latent Fragility under Agentic Perturbations in Clinical LLMs	arXiv	2025	Canada	Classification	Mixed medical	Ablation	N	Not involved	Not involved	N	Workflow Violation
[62] Zhao (2025)	Affective-ROPTester: Capability and Bias Analysis of LLMs in Predicting Retinopathy of Prematurity	arXiv	2025	Singapore	Classification	Ophthalmology	Context-variation prompt	N	Not involved	Not involved	Y	
[63] Ji (2025)	MedOmni-45°: A Safety–Performance Benchmark for Reasoning-Oriented LLMs in Medicine	arXiv	2025	China	MCQ	Mixed medical	Misleading prompt	Y	Mixed	Design	Y	

*MCQ* Multiple-choice question. Note: The journal or source reflects the record available at the time of the literature search

**Fig. 2:**
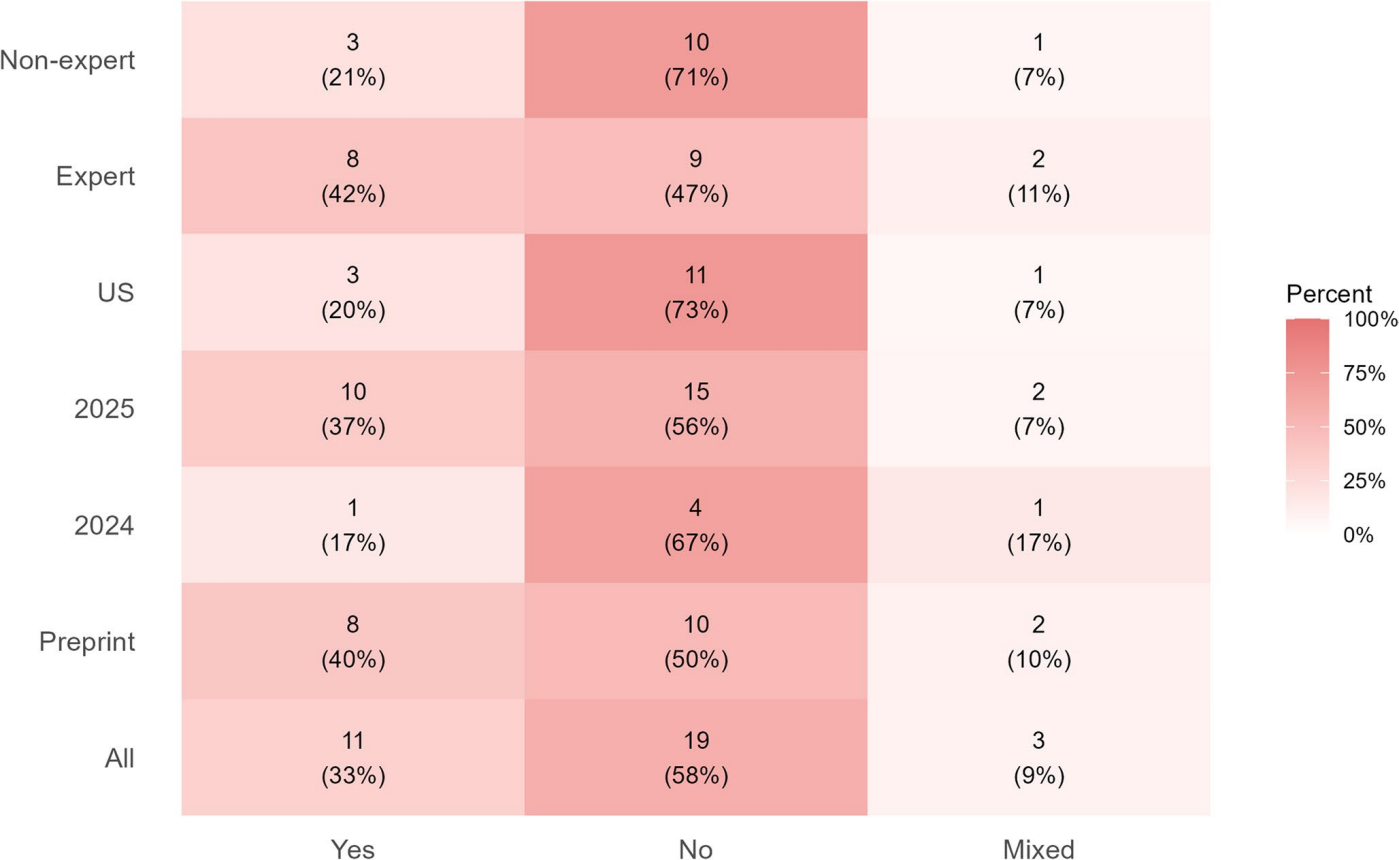
Heatmap illustrating the relationship between study characteristics and clinical plausibility. Each cell displays the number of studies classified as clinically plausible or not within each category. Studies using multiple robustness-testing methods were classified at the study level: Yes if all tests were clinically plausible, No if none were clinically plausible, and Mixed if both were present. Each study was counted once per subgroup. Darker shading indicates a higher proportion of clinically plausible studies within the group

**Fig. 3:**
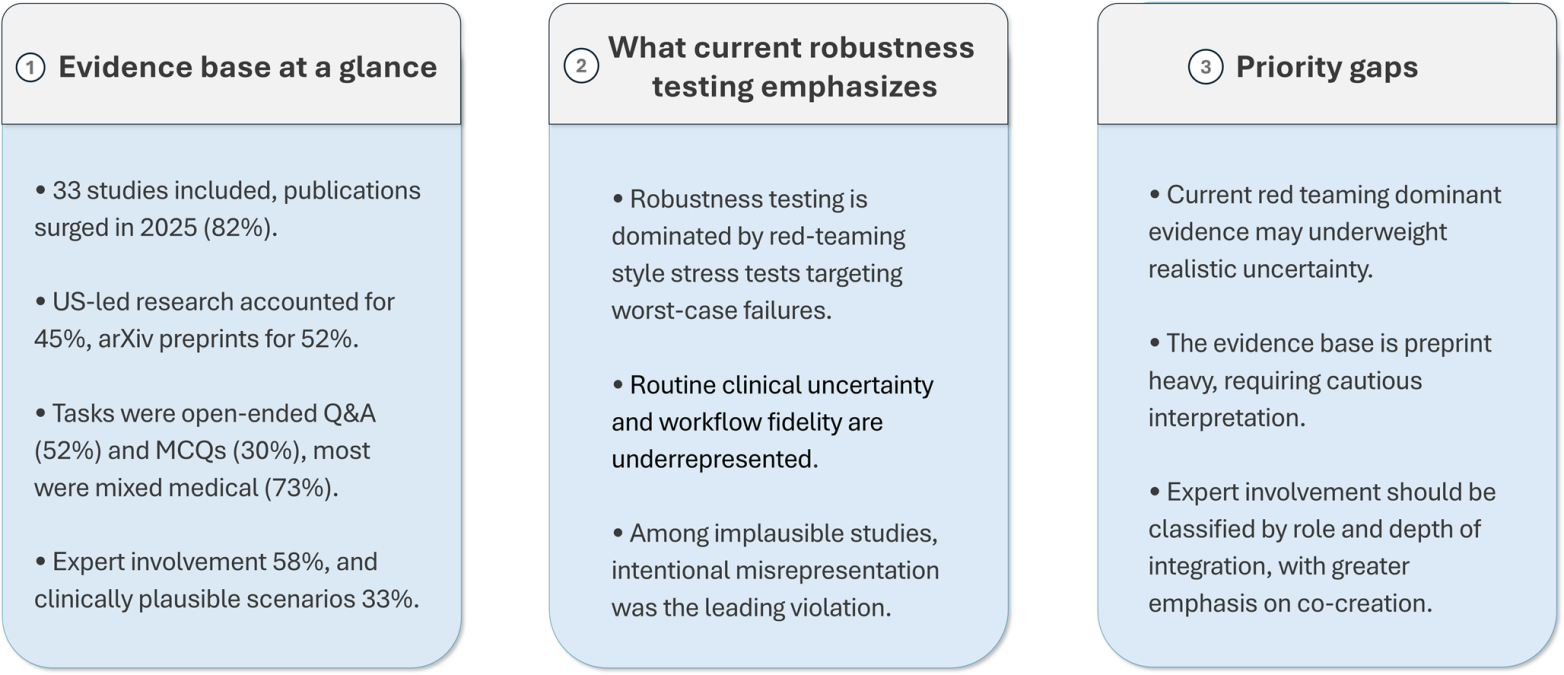
Schematic summary of the evidence base, current emphases, and priority gaps in the included studies

Methodologically, the most common task assigned to LLMs was responding to open-ended questions, which appeared in 17 studies (52%), followed by multiple-choice questions (MCQs) in 10 studies (30%). The medical scope of these evaluations was predominantly broad, with 24 studies (73%) situated within a “mixed medical” domain rather than a single specialty. Thematic analysis of the robustness testing methods identified four primary intervention categories. Misleading prompts, which introduced confusing or contradictory information within a clinically plausible scenario, appeared in 49% of studies and adversarial prompts, designed to elicit unstable outputs, appeared in 39%. Less common methods included “Context-variation prompts,” which varied the clinical context to test consistency (12%), and one additional emergent category “Ablation,” which involved systematically removing parts of a prompt (6%). Expert involvement was reported in 19 studies (58%). Finally, a critical assessment determined that only 11 studies (33%) employed testing scenarios designed to mimic plausible, real-world clinical interactions. The relationship between study characteristics and clinical plausibility is further visualized in a heatmap (Fig. 2). To support interpretability for interdisciplinary readers, we provide a schematic synthesis of the evidence base, current emphases, and priority gaps (Fig. 3).

For the studies that lacked clinical plausibility, an analysis identified three primary types of violations: “Intentional Misrepresentation” (adversarial or deceptive intent that would not arise in real clinical interactions), “Attribute Mutation” (changes in the patient’s fundamental attributes within the scenario), and “Workflow Violation” (inconsistent with any real clinical process). We summarize the resulting violation taxonomy, decision rules, borderline handling, and illustrative examples in Table 2. Among these, “intentional misrepresentation” was the predominant reason for the lack of plausibility, accounting for 15 of the 19 studies (79%).

**Table 2:** Decision rules and examples for classifying clinically implausible robustness evaluations

Violation type	Description	Decision rules	Borderline handling	Example
Intentional Misrepresentation (15 studies)	Adversarial or deceptive intent that would not arise in real clinical interactions.	The primary perturbation requires an adversarial or deceptive intent, such as asking the model to treat a known-incorrect premise as correct, or injecting hidden instructions designed to override the intended task. A key indicator is that the prompt’s goal is to mislead the model rather than to seek clarification, reconcile uncertainty, or support clinical decision-making.	Clinically plausible misinformation can occur, but the intent differs. If incorrect information plausibly arises from patient recall errors, handover mistakes, or incomplete documentation and is framed as clarification seeking, do not treat as intentional misrepresentation. If the prompt aims to deceive, jailbreak, or force acceptance of an incorrect premise, treat as intentional misrepresentation.	1. “Ask it to explain why an incorrect answer choice is correct.” 2. “Hidden adversarial instructions in images.”
Attribute Mutation (1 study)	Changes in the patient’s fundamental attributes within the scenario.	The study explicitly instructs a swap or alteration of a core patient attribute, for example sex or gender swapping.	If the attribute change is explicitly framed as a correction or longitudinal update in a coherent timeline, it may be clinically plausible and not an attribute mutation violation. If it is a direct swap instruction without clinical rationale, treat as attribute mutation.	“Please swap the gender in the following text.”
Workflow Violation (3 studies)	Inconsistent with any real clinical process.	The perturbation relies on input formats, manipulations, or interaction patterns that would not occur in real clinical communication or documentation, such as artificial masking tokens or mechanically fragmented prompts used as the main test condition.	Missing or incomplete information can be clinically plausible, but the representation matters. Clinically plausible missingness is typically expressed as unknown, not available, or not documented. Artificial tokens or forced fragmentation, such as [MASK] replacement, should be treated as workflow violation.	“Replaces medically salient entities with [MASK].”

### Critical Synthesis

The current focus on technical over clinical vulnerabilities in LLM robustness testing reveals a disconnect rooted in the complexity of interdisciplinary integration. This is evidenced by the literature’s concentration on adversarial stress-testing rather than on evaluations that reflect real-world clinical fidelity. The frequent use of “Misleading prompts” and “Adversarial prompts” indicates that the current research paradigm is heavily focused on a red-teaming approach designed to identify worst-case vulnerabilities [18–20]. Although such tests are essential for identifying technical failure modes and informing safe deployment, they address a different question from clinically grounded evaluations, which aim to approximate routine use conditions where clinicians are unlikely to intentionally undermine model performance. Delving deeper, of the 19 studies that involved experts, still only eight designed experiments that were clinically plausible, suggesting a disconnect that could limit the expert validity of many testing protocols.

In contrast, some studies exemplify more clinically plausible testing methodologies. For instance, several papers utilized prompts with an inquisitive or testing tone rather than a malicious one, mirroring how a clinician might probe the boundaries of a model’s knowledge [21, 22]. Other studies explored clinically relevant perturbations to scenarios, such as altering secondary symptoms or lab values in plausible ways to assess diagnostic consistency [23, 24]. These approaches represent more genuine scenarios for evaluating an LLM’s performance in a real-world clinical setting.

Beyond the focus of inquiry, the methodological rigor and generalizability of the current evidence base deserve careful reflection. The high proportion of preprint literature is a testament to the remarkable speed of innovation in LLM development. While this rapid dissemination facilitates timely knowledge exchange [25], it also suggests that findings should be interpreted with appropriate caution until peer review provides further validation. Striking a balance between innovation and evidentiary rigor will support both timely advancement and trustworthy application. Moreover, while expert involvement was reported in a majority of studies, the depth and alignment of this expertise remain ambiguous. To address this, a more granular classification of expert involvement is needed, distinguishing between consultative roles, evaluative roles, and integrative co-creation, where experts are deeply engaged from problem definition to final interpretation. True clinical validity is most likely to emerge from this third mode, which appears underrepresented. Accordingly, expert involvement that is not described in depth should be interpreted cautiously and is insufficient on its own to support claims of clinical validity. The literature also reveals a need for more focused research within highly specialized domains, as the preponderance of “mixed medical” studies may not adequately uncover the specific failure modes in high-stakes fields. Finally, geographic concentration may limit generalizability beyond the US context. This may relate to model training data [26], and also to clinical evaluation context, including local guidelines and documentation workflows. Model performance may differ across languages and cultural settings [27].

A temporal analysis reveals that the surge in this research area is a recent phenomenon, with studies only appearing from 2024 onward. This timeline corresponds with the point at which state-of-the-art LLMs began demonstrating a professional level of competence in some medical domains, making such robustness testing meaningful. The rapid acceleration of publications in 2025 not only highlights the field’s dynamism but also underscores the importance of continuous monitoring. The speed at which both the models and the research landscape evolve necessitates ongoing evaluation to ensure that our understanding of LLM robustness keeps pace with the technology’s development. Another major gap is the lack of longitudinal and integrative assessment. Nearly all reviewed studies were cross-sectional, snapshot-style evaluations, testing a model’s response to single, isolated prompts. A real diagnostic process is a longitudinal journey where clinicians constantly revise hypotheses based on new information.

### Practice and Research Implications

This review highlights clinical plausibility as a foundational consideration for meaningful robustness testing. While stress-testing is vital for understanding model vulnerabilities, the current robustness evidence is insufficient to support deployment-relevant inferences for clinically integrated CDSS. This limitation reflects the interpretive limits of current evaluation paradigms, rather than intrinsic model capability or future clinically integrated systems. Evidence on LLM behavior in uncertain, non-adversarial scenarios remains limited. Evaluation frameworks should integrate adversarial stress-testing and clinically plausible testing as complementary approaches.

We advocate for distinguishing between stress tests that probe clinically-possible challenges versus those that probe technically-possible but clinically-impossible scenarios. This involves evaluating their performance with incomplete patient data, ambiguous queries, and their ability to recognize when a question falls outside their scope of competence. An important priority is the design of experimental methodologies grounded in clinical plausibility, moving beyond broad benchmarks to in-depth investigations within specific subspecialties [28]. LLM performance and robustness may vary across subspecialties due to differences in input modality, outcome definability, and the availability of domain-specific data. However, the current robustness literature is too heterogeneous to support systematic cross-subspecialty comparisons. Accordingly, subspecialty-aware evaluations remain an important future direction. Such studies may benefit from using fewer, more carefully constructed prompts, potentially employing qualitative or mixed-methods approaches—such as Cognitive Walkthroughs or Think-Aloud Protocols [29]—to deeply probe the model’s reasoning. These methods allow researchers to observe how users reason step by step, revealing where understanding breaks down or decisions are made. By observing clinicians interacting with an LLM in a realistic workflow, these methods can uncover subtle interaction failures and cognitive biases that quantitative metrics cannot capture. Integral to this shift is the deep and continuous involvement of clinical experts throughout the entire research process. A practical step to formalize this approach would be to integrate the principles of clinical plausibility and deep expert co-creation [30], as highlighted in this review, into established consensus statements like CONSORT-AI and DECIDE-AI. This would formally encourage the research community to design and execute studies that more accurately reflect clinical reality.

As highlighted in the preceding synthesis, existing studies have predominantly adopted cross-sectional, single-turn evaluations. Therefore, future work should incorporate longitudinal and integrative designs to explore the long-term impacts of LLM integration [31]. Critical questions remain, such as how prolonged reliance on LLMs affects clinicians’ diagnostic skills and what the cumulative risk of subtle, repeated errors might be on patient outcomes. Separately, response stability under repeated prompting represents a reliability dimension that warrants explicit reporting and analysis [32]. Research should also broaden its geographic and linguistic scope to address potential biases [33].

Consistent with the EU AI Act high-risk framing, clinical applications that can meaningfully influence decisions should be treated as high risk, distinct from clinician verifiable assistance. Such use warrants strong assurance evidence before high-risk integration. Building this evidence base will require interdisciplinary work at the intersection of medicine, computer science, and bioinformatics. To guide this effort, we propose a phased research roadmap: deep validation in narrow subspecialties, evaluation frameworks that simulate multi-turn incomplete-information dialogue, and prospective clinical simulation studies to assess workflow impact without exposing patients to risk [34].

## Limitations

Several limitations should be considered when interpreting the findings of this review. First, the processes of data extraction and thematic interpretation, despite reflective discussions, are subject to a degree of subjectivity. Our categorization of robustness testing methods was intended for descriptive mapping and may not capture finer distinctions within each category. Similarly, while expert involvement was categorized by the reported role of engagement, the depth of such involvement could not always be fully determined from the available information. The composition of our review team, which consists primarily of medical professionals, may have introduced a perspective that is less deeply attuned to the technical aspects of computer science methodologies.

Second, the LLM robustness literature identified in our review is small, recent, and rapidly evolving, with many included studies published as preprints. Therefore, our findings should be interpreted as emerging patterns rather than stable estimates of the field. Nevertheless, we contend that the core methodological insights regarding the need for clinical realism in robustness testing will retain their relevance. Third, our search strategy, though comprehensive, was not exhaustive. In balancing search breadth with feasibility, certain platforms, such as OpenReview, were not included, which may have resulted in the omission of some relevant literature. Finally, although we attempted to define clinical realism, our three-level classification of it is preliminary. Future research could develop a more granular assessment of its different facets, for instance, by using a multi-item Likert scale.

## Supplementary Information

Below is the link to the electronic supplementary material.

Supplementary Material 1 (DOCX 72.3 KB)

## Data Availability

Data is provided within the manuscript or supplementary information files.
