# Supplementary material for: Clinical Plausibility in Large Language Model Robustness Testing for Medicine: A Scoping Review
Source: J Med Syst. 2026 May 11;50(1):77. doi: 10.1007/s10916-026-02405-1 (PMC13161009; doi:10.1007/s10916-026-02405-1)
Supplement: Supplementary file 1 — Supplementary Material 1 (DOCX 72.3 KB) [file 10916_2026_2405_MOESM1_ESM.docx]

**Table of Content**

**Method S1. Deviation from the registered protocol**

**Method S2. Preferred Reporting Items for Systematic reviews and Meta-Analyses extension for Scoping Reviews (PRISMA-ScR) Checklist**

**Method S3. Search keywords**

**Method S4. R scripts for arXiv and medRxiv search**

**Result S1. Excluded full-text articles and corresponding reasons for exclusion**

**Method S1. Deviation from the registered protocol**

The conduct of this scoping review adhered closely to the preregistered protocol, with a few minor deviations made during the research process to refine the scope and enhance analytical focus. First, the eligibility criteria were sharpened to increase specificity. We refined the exclusion criteria to remove studies focused on simple information summarization tasks (e.g., generating discharge summaries) that lacked a direct inferential or decision-support component. Additionally, we excluded studies that did not use a discernible comparative benchmark to ensure a minimum standard of evaluative rigor. Conversely, the language criteria were broadened from including non-English studies only if they had an English abstract to imposing no language restrictions. These adjustments were made post-hoc to more precisely target the literature on LLMs used in high-stakes clinical reasoning contexts. Second, the search strategy was slightly optimized; while the core concepts remained unchanged, the final search query used a more focused set of keywords for robustness testing, omitting broader terms like 'validation' and 'evaluation' to improve the precision of the search results. Finally, the most significant evolution from the protocol occurred during the data charting and synthesis phase. The initial, broader concept of assessing "clinical authenticity" was operationalized into a key, novel variable: a binary classification of whether a test mimicked a "plausible clinical scenario" (Yes/No). This simplification provided a clear and actionable framework that directly addressed the review's central research question regarding the alignment of testing methodologies with real-world clinical practice.

**Method S2. Preferred Reporting Items for Systematic reviews and Meta-Analyses extension for Scoping Reviews (PRISMA-ScR) Checklist**

| **SECTION** | **ITEM** | **PRISMA-ScR CHECKLIST ITEM** | **REPORTED ON PAGE #** |
| --- | --- | --- | --- |
| **TITLE** | | | |
| Title | 1 | Identify the report as a scoping review. | Title |
| **ABSTRACT** | | | |
| Structured summary | 2 | Provide a structured summary that includes (as applicable): background, objectives, eligibility criteria, sources of evidence, charting methods, results, and conclusions that relate to the review questions and objectives. | Abstract |
| **INTRODUCTION** | | | |
| Rationale | 3 | Describe the rationale for the review in the context of what is already known. Explain why the review questions/objectives lend themselves to a scoping review approach. | Rationale/Question |
| Objectives | 4 | Provide an explicit statement of the questions and objectives being addressed with reference to their key elements (e.g., population or participants, concepts, and context) or other relevant key elements used to conceptualize the review questions and/or objectives. | Rationale/Question |
| **METHODS** | | | |
| Protocol and registration | 5 | Indicate whether a review protocol exists; state if and where it can be accessed (e.g., a Web address); and if available, provide registration information, including the registration number. | Method > Protocol and Registration |
| Eligibility criteria | 6 | Specify characteristics of the sources of evidence used as eligibility criteria (e.g., years considered, language, and publication status), and provide a rationale. | Method > Eligibility Criteria |
| Information sources* | 7 | Describe all information sources in the search (e.g., databases with dates of coverage and contact with authors to identify additional sources), as well as the date the most recent search was executed. | Method > Information Sources and Search Strategy |
| Search | 8 | Present the full electronic search strategy for at least 1 database, including any limits used, such that it could be repeated. | Method > Information Sources and Search Strategy |
| Selection of sources of evidence† | 9 | State the process for selecting sources of evidence (i.e., screening and eligibility) included in the scoping review. | Method > Selection and Data Charting Process |
| Data charting process‡ | 10 | Describe the methods of charting data from the included sources of evidence (e.g., calibrated forms or forms that have been tested by the team before their use, and whether data charting was done independently or in duplicate) and any processes for obtaining and confirming data from investigators. | Method > Selection and Data Charting Process |
| Data items | 11 | List and define all variables for which data were sought and any assumptions and simplifications made. | Method > Selection and Data Charting Process |
| Critical appraisal of individual sources of evidence§ | 12 | If done, provide a rationale for conducting a critical appraisal of included sources of evidence; describe the methods used and how this information was used in any data synthesis (if appropriate). | Method > Synthesis of Results (States that a formal quality appraisal was not performed and provides the rationale.) |
| Synthesis of results | 13 | Describe the methods of handling and summarizing the data that were charted. | Method > Synthesis of Results |
| **RESULTS** | | | |
| Selection of sources of evidence | 14 | Give numbers of sources of evidence screened, assessed for eligibility, and included in the review, with reasons for exclusions at each stage, ideally using a flow diagram. | Results (Paragraph 1) |
| Characteristics of sources of evidence | 15 | For each source of evidence, present characteristics for which data were charted and provide the citations. | Results (Paragraph 2 and 3) |
| Critical appraisal within sources of evidence | 16 | If done, present data on critical appraisal of included sources of evidence (see item 12). | Method > Synthesis of Results (States that this was not performed.) |
| Results of individual sources of evidence | 17 | For each included source of evidence, present the relevant data that were charted that relate to the review questions and objectives. | Results (The data is presented in aggregate, which is appropriate for a scoping review.) |
| Synthesis of results | 18 | Summarize and/or present the charting results as they relate to the review questions and objectives. | Results; Critical Synthesis |
| **DISCUSSION** | | | |
| Summary of evidence | 19 | Summarize the main results (including an overview of concepts, themes, and types of evidence available), link to the review questions and objectives, and consider the relevance to key groups. | Critical Synthesis; Practice and Research Implications |
| Limitations | 20 | Discuss the limitations of the scoping review process. | Limitations |
| Conclusions | 21 | Provide a general interpretation of the results with respect to the review questions and objectives, as well as potential implications and/or next steps. | Practice and Research Implications |
| **FUNDING** | | | |
| Funding | 22 | Describe sources of funding for the included sources of evidence, as well as sources of funding for the scoping review. Describe the role of the funders of the scoping review. | Funding |

**Method S3. Search keywords**

[PubMed]

(("large language model"[tiab] OR LLM[tiab] OR "generative pre-trained transformer"[tiab] OR GPT[tiab] OR ChatGPT[tiab] OR Gemini[tiab] OR Claude[tiab])

AND

(medical[tiab] OR clinical[tiab] OR healthcare[tiab] OR "patient care"[tiab] OR diagnosis[tiab] OR treatment[tiab])

AND

(reliability[tiab] OR robustness[tiab] OR "adversarial*"[tiab] OR "prompt*"[tiab] OR "red team*"[tiab] OR "stress test*"[tiab] OR "uncertainty"[tiab]))

AND ("2023/01/01"[Date - Publication] : "2025/09/23"[Date - Publication])

[IEEE Xplore]

("All Metadata":"large language model" OR "All Metadata":LLM OR "All Metadata":"generative pre-trained transformer" OR "All Metadata":GPT OR "All Metadata":ChatGPT OR "All Metadata":Gemini OR "All Metadata":Claude) AND ("All Metadata":medical OR "All Metadata":clinical OR "All Metadata":healthcare OR "All Metadata":"patient care" OR "All Metadata":diagnosis OR "All Metadata":treatment) AND ("All Metadata":reliability OR "All Metadata":robustness OR "All Metadata":"adversarial*" OR "All Metadata":"prompt*" OR "All Metadata":"red team*" OR "All Metadata":"stress test*" OR "All Metadata":"uncertainty")

Filters Applied: JournalsEarly Access Articles01/01/2023 - 09/23/2025

[Embase]

('large language model':ti,ab,kw OR llm:ti,ab,kw OR 'generative pre-trained transformer':ti,ab,kw OR gpt:ti,ab,kw OR chatgpt:ti,ab,kw OR gemini:ti,ab,kw OR claude:ti,ab,kw) AND (medical:ti,ab,kw OR clinical:ti,ab,kw OR healthcare:ti,ab,kw OR 'patient care':ti,ab,kw OR diagnosis:ti,ab,kw OR treatment:ti,ab,kw) AND (reliability:ti,ab,kw OR robustness:ti,ab,kw OR adversarial*:ti,ab,kw OR prompt*:ti,ab,kw OR 'red team*':ti,ab,kw OR 'stress test*':ti,ab,kw OR uncertainty:ti,ab,kw)

[WOS core]

((((TS=("large language model" OR LLM OR "generative pre-trained transformer" OR GPT OR ChatGPT OR Gemini OR Claude)) AND TS=(medical OR clinical OR healthcare OR "patient care" OR diagnosis OR treatment)) AND TS=(reliability OR robustness OR adversarial* OR prompt* OR "red team*" OR "stress test*" OR uncertainty))) AND PY=(2023-2026)

[ACM DL]

[[Abstract: "large language model"] OR [Abstract: llm] OR [Abstract: "generative pre-trained transformer"] OR [Abstract: gpt] OR [Abstract: chatgpt] OR [Abstract: gemini] OR [Abstract: claude]] AND [[Abstract: medical] OR [Abstract: clinical] OR [Abstract: healthcare] OR [Abstract: "patient care"] OR [Abstract: diagnosis] OR [Abstract: treatment]] AND [[Abstract: reliability] OR [Abstract: robustness] OR [Abstract: adversarial*] OR [Abstract: prompt*] OR [Abstract: "red team*"] OR [Abstract: "stress test*"] OR [Abstract: uncertainty]] AND [E-Publication Date: (01/01/2023 TO 12/31/2025)]

[[Title: "large language model"] OR [Title: llm] OR [Title: "generative pre-trained transformer"] OR [Title: gpt] OR [Title: chatgpt] OR [Title: gemini] OR [Title: claude]] AND [[Title: medical] OR [Title: clinical] OR [Title: healthcare] OR [Title: "patient care"] OR [Title: diagnosis] OR [Title: treatment]] AND [[Title: reliability] OR [Title: robustness] OR [Title: adversarial*] OR [Title: prompt*] OR [Title: "red team*"] OR [Title: "stress test*"] OR [Title: uncertainty]] AND [E-Publication Date: (01/01/2023 TO 12/31/2025)]

**Method S4. R scripts for arXiv and medRxiv search**

##aRxiv##

library(aRxiv)

library(dplyr)

library(lubridate)

A <- '(ti:"large language model" OR ti:LLM OR ti:"generative pre-trained transformer" OR ti:GPT OR ti:ChatGPT OR ti:Gemini OR ti:Claude OR abs:"large language model" OR abs:LLM OR abs:"generative pre-trained transformer" OR abs:GPT OR abs:ChatGPT OR abs:Gemini OR abs:Claude)'

B <- '(ti:medical OR ti:clinical OR ti:healthcare OR ti:"patient care" OR ti:diagnosis OR ti:treatment OR abs:medical OR abs:clinical OR abs:healthcare OR abs:"patient care" OR abs:diagnosis OR abs:treatment)'

C <- '(ti:reliability OR ti:robustness OR ti:adversarial* OR ti:prompt* OR ti:"red team*" OR ti:"stress test*" OR ti:uncertainty OR abs:reliability OR abs:robustness OR abs:adversarial* OR abs:prompt* OR abs:"red team*" OR abs:"stress test*" OR abs:uncertainty)'

query_base <- paste(A, B, C, sep = " AND ")

start_date <- ymd("2023-01-01")

end_date <- ymd("2025-09-23")

date_query_total <- paste0('submittedDate:[',

format(start_date, "%Y%m%d%H%M"),

' TO ',

format(end_date, "%Y%m%d%H%M"),

']')

query_for_count <- paste(query_base, date_query_total, sep = " AND ")

n_expected <- arxiv_count(query_for_count)

date_sequence <- seq(from = floor_date(start_date, "month"),

to = floor_date(end_date, "month"),

by = "month")

all_results_list <- list()

for (i in 1:length(date_sequence)) {

month_start <- date_sequence[i]

month_end <- ceiling_date(month_start, "month") - days(1)

if (month_end > end_date) {

month_end <- end_date

}

start_str <- format(month_start, "%Y%m%d0000")

end_str <- format(month_end, "%Y%m%d2359")

date_query_monthly <- paste0("submittedDate:[", start_str, " TO ", end_str, "]")

query_monthly <- paste(query_base, date_query_monthly, sep = " AND ")

res_monthly <- arxiv_search(

query = query_monthly,

limit = 5000,

sort_by = "submitted",

ascending = FALSE

)

res_arxiv_final <- bind_rows(all_results_list)

if (is.data.frame(res_monthly) && nrow(res_monthly) > 0) {

all_results_list[[format(month_start, "%Y-%m")]] <- res_monthly

} else {

cat("No data in this month.\n")

}

}

write.csv(res_arxiv_final, "arxiv.csv", row.names = FALSE)

##MedrXiv##

install.packages("remotes")

remotes::install_github("ropensci/medrxivr")

library(medrxivr)

preprint_data <- mx_snapshot()

topic_A <- c("large language model", "LLM", "generative pre-trained transformer", "GPT", "ChatGPT", "Gemini", "Claude")

topic_B <- c("medical", "clinical", "healthcare", "patient care", "diagnosis", "treatment")

topic_C <- c("reliability", "robustness", "adversarial", "prompt", "red team", "stress test", "uncertainty")

res_medrxiv <- mx_search(

data = preprint_data,

query = list(topic_A, topic_B, topic_C),

fields = c("title", "abstract"),

from_date = "2023-01-01",

to_date = "2025-09-23",

auto_caps = TRUE,

deduplicate = TRUE,

report = TRUE

)

write.csv(res_medrxiv, "medrxiv.csv", row.names = FALSE)

**Result S1. Excluded full-text articles and corresponding reasons for exclusion**

[1–16] : Wrong study design

[17–24]: Repetition

[25–42]: Wrong study outcome

1. Hao W-R, Chen C-C, Chen K, Li L-C, Chiu C-C, Yang T-Y, Jong H-C, Yang H-C, Huang C-W, Liu J-C, Li Y-CJ (2025) ChatGPT Performance Deteriorated in Patients with Comorbidities When Providing Cardiological Therapeutic Consultations. Healthcare (Basel) 13:1598. https://doi.org/10.3390/healthcare13131598

2. Schramm S, Preis S, Metz M-C, Jung K, Schmitz-Koep B, Zimmer C, Wiestler B, Hedderich DM, Kim SH (2025) Impact of Multimodal Prompt Elements on Diagnostic Performance of GPT-4V in Challenging Brain MRI Cases. Radiology 314:e240689. https://doi.org/10.1148/radiol.240689

3. Kliem PSC, Fisch U, Baumann SM, Berger S, Amacher SA, Hunziker S, Sutter R (2025) The impact of prompting on ChatGPT’s adherence to status epilepticus treatment guidelines. Sci Rep 15:31712. https://doi.org/10.1038/s41598-025-16902-9

4. Skittle C, Bonifacino E, McQuade CN (2025) Medical language matters: impact of clinical summary composition on a generative artificial intelligence’s diagnostic accuracy. Diagnosis (Berl) 12:277–281. https://doi.org/10.1515/dx-2024-0167

5. Kim SH, Ziegelmayer S, Busch F, Mertens CJ, Keicher M, Adams LC, Bressem KK, Braren R, Makowski MR, Kirschke JS, Hedderich DM, Wiestler B (2025) LLM Reasoning Does Not Protect Against Clinical Cognitive Biases - An Evaluation Using BiasMedQA. 2025.06.22.25330078

6. Heston TF (2023) Evaluating Risk Progression in Mental Health Chatbots Using Escalating Prompts. 2023.09.10.23295321

7. Peng B, Chen K, Niu Q, Bi Z, Liu M, Feng P, Wang T, Yan LKQ, Wen Y, Zhang Y, Yin CH (2025) Jailbreaking and Mitigation of Vulnerabilities in Large Language Models

8. BN S, Shing H-C, Xu L, Strong M, Burnsky J, Ofor J, Mason JR, Chen S, Srinivasan S, Shivade C, Moriarty J, Cohen JP (2025) Fact-Controlled Diagnosis of Hallucinations in Medical Text Summarization

9. Jiang E, Xu C, Singh N, Singh G (2025) Misaligning Reasoning with Answers -- A Framework for Assessing LLM CoT Robustness

10. Draelos RL, Afreen S, Blasko B, Brazile TL, Chase N, Desai DP, Evert J, Gardner HL, Herrmann L, House AV, Kass S, Kavan M, Khemani K, Koire A, McDonald LM, Rabeeah Z, Shah A (2025) Large language models provide unsafe answers to patient-posed medical questions

11. Hussain A, Zhao P, Vincent N (2025) An Audit and Analysis of LLM-Assisted Health Misinformation Jailbreaks Against LLMs

12. Ruan W, Yi X, Huang X (2021) Adversarial Robustness of Deep Learning: Theory, Algorithms, and Applications

13. Zhang Y, Chen K, Gao J, Cui R, Wang R, Wang L, Zhang T (2025) Towards Action Hijacking of Large Language Model-based Agent

14. Chang CT, Farah H, Gui H, Rezaei SJ, Bou-Khalil C, Park Y-J, Swaminathan A, Omiye JA, Kolluri A, Chaurasia A, Lozano A, Heiman A, Jia AS, Kaushal A, Jia A, Iacovelli A, Yang A, Salles A, Singhal A, Narasimhan B, Belai B, Jacobson BH, Li B, Poe CH, Sanghera C, Zheng C, Messer C, Kettud DV, Pandya D, Kaur D, Hla D, Dindoust D, Moehrle D, Ross D, Chou E, Lin E, Haredasht FN, Cheng G, Gao I, Chang J, Silberg J, Fries JA, Xu J, Jamison J, Tamaresis JS, Chen JH, Lazaro J, Banda JM, Lee JJ, Matthys KE, Steffner KR, Tian L, Pegolotti L, Srinivasan M, Manimaran M, Schwede M, Zhang M, Nguyen M, Fathzadeh M, Zhao Q, Bajra R, Khurana R, Azam R, Bartlett R, Truong ST, Fleming SL, Raj S, Behr S, Onyeka S, Muppidi S, Bandali T, Eulalio TY, Chen W, Zhou X, Ding Y, Cui Y, Tan Y, Liu Y, Shah N, Daneshjou R (2025) Red teaming ChatGPT in medicine to yield real-world insights on model behavior. npj Digit Med 8:149. https://doi.org/10.1038/s41746-025-01542-0

15. Sorin V, Collins JD, Bratt AK, Kusmirek JE, Mugu VK, Kline TL, Butler CL, Wood NG, Cook CJ, Korfiatis P (2025) Evaluating prompt and data perturbation sensitivity in large language models for radiology reports classification. JAMIA Open 8:ooaf073. https://doi.org/10.1093/jamiaopen/ooaf073

16. Han T, Nebelung S, Khader F, Wang T, Müller-Franzes G, Kuhl C, Försch S, Kleesiek J, Haarburger C, Bressem KK, Kather JN, Truhn D (2024) Medical large language models are susceptible to targeted misinformation attacks. npj Digit Med 7:288. https://doi.org/10.1038/s41746-024-01282-7

17. Omar M, Sorin V, Collins JD, Reich D, Freeman R, Gavin N, Charney A, Stump L, Bragazzi NL, Nadkarni GN, Klang E (2025) Large Language Models Are Highly Vulnerable to Adversarial Hallucination Attacks in Clinical Decision Support: A Multi-Model Assurance Analysis. 2025.03.18.25324184

18. Clusmann J, Schulz SJK, Ferber D, Wiest IC, Fernandez A, Eckstein M, Lange F, Reitsam NG, Kellers F, Schmitt M, Neidlinger P, Koop P-H, Schneider CV, Truhn D, Roth W, Jesinghaus M, Kather JN, Foersch S (2024) A pen mark is all you need - Incidental prompt injection attacks on Vision Language Models in real-life histopathology. 2024.12.11.24318840

19. Zada T, Tam N, Barnard F, Sittert MV, Bhat V, Rambhatla S (2023) Medical Misinformation in AI-Assisted Self-Diagnosis: Development of a Method (EvalPrompt) for Analyzing Large Language Models. arXiv preprint

20. Safrai M, Azaria A (2023) Performance of ChatGPT-3.5 and GPT-4 on the United States Medical Licensing Examination With and Without Distractions

21. Clusmann J, Ferber D, Wiest IC, Schneider CV, Brinker TJ, Foersch S, Truhn D, Kather JN (2024) Prompt Injection Attacks on Large Language Models in Oncology

22. Chen S, Gao M, Sasse K, Hartvigsen T, Anthony B, Fan L, Aerts H, Gallifant J, Bitterman D (2024) Wait, but Tylenol is Acetaminophen... Investigating and Improving Language Models’ Ability to Resist Requests for Misinformation

23. Vishwanath K, Alyakin A, Ghosh M, Lee JV, Alber DA, Sangwon KL, Kondziolka D, Oermann EK (2025) Evaluating the performance and fragility of large language models on the self-assessment for neurological surgeons

24. Gallifant J, Chen S, Moreira P, Munch N, Gao M, Pond J, Celi LA, Aerts H, Hartvigsen T, Bitterman D (2024) Language Models are Surprisingly Fragile to Drug Names in Biomedical Benchmarks

25. Wang J, Hu X, Hou W, Chen H, Zheng R, Wang Y, Yang L, Huang H, Ye W, Geng X, Jiao B, Zhang Y, Xie X (2023) On the Robustness of ChatGPT: An Adversarial and Out-of-distribution Perspective

26. Deshpande A, Murahari V, Rajpurohit T, Kalyan A, Narasimhan K (2023) Toxicity in ChatGPT: Analyzing Persona-assigned Language Models

27. Wang B, Chen W, Pei H, Xie C, Kang M, Zhang C, Xu C, Xiong Z, Dutta R, Schaeffer R, Truong ST, Arora S, Mazeika M, Hendrycks D, Lin Z, Cheng Y, Koyejo S, Song D, Li B (2024) DecodingTrust: A Comprehensive Assessment of Trustworthiness in GPT Models

28. Bhardwaj R, Poria S (2023) Language Model Unalignment: Parametric Red-Teaming to Expose Hidden Harms and Biases

29. Zhang X, Li J, Chu W, Hai J, Xu R, Yang Y, Guan S, Xu J, Cui P (2024) On the Out-Of-Distribution Generalization of Multimodal Large Language Models

30. Poulain R, Fayyaz H, Beheshti R (2024) Bias patterns in the application of LLMs for clinical decision support: A comprehensive study

31. Chen Z, Xiang Z, Xiao C, Song D, Li B (2024) AgentPoison: Red-teaming LLM Agents via Poisoning Memory or Knowledge Bases

32. Arroyo AMC, Munnangi M, Sun J, Zhang KYC, McInerney DJ, Wallace BC, Amir S (2024) Open (Clinical) LLMs are Sensitive to Instruction Phrasings

33. Sepehri MS, Fabian Z, Soltanolkotabi M, Soltanolkotabi M (2025) MediConfusion: Can you trust your AI radiologist? Probing the reliability of multimodal medical foundation models

34. Kumar V, Ntoutsi E, Rajawat PS, Medda G, Recupero DR (2024) Unlocking LLMs: Addressing Scarce Data and Bias Challenges in Mental Health

35. Zhang H, Lou Q, Wang Y (2025) Towards Safe AI Clinicians: A Comprehensive Study on Large Language Model Jailbreaking in Healthcare

36. Pandit S, Xu J, Hong J, Wang Z, Chen T, Xu K, Ding Y (2025) MedHallu: A Comprehensive Benchmark for Detecting Medical Hallucinations in Large Language Models

37. Fanous A, Goldberg J, Agarwal AA, Lin J, Zhou A, Daneshjou R, Koyejo S (2025) SycEval: Evaluating LLM Sycophancy

38. Zou J, Li Q, Lian C, Liu L, Yan X, Wang S, Qin J (2025) CorBenchX: Large-Scale Chest X-Ray Error Dataset and Vision-Language Model Benchmark for Report Error Correction

39. Sato M (2025) Triggering Hallucinations in LLMs: A Quantitative Study of Prompt-Induced Hallucination in Large Language Models

40. Corbeil J-P, Kim M, Sordoni A, Beaulieu F, Vozila P (2025) Medical Red Teaming Protocol of Language Models: On the Importance of User Perspectives in Healthcare Settings

41. Iglesia ID la, Goenaga I, Ramirez-Romero J, Villa-Gonzalez JM, Goikoetxea J, Barrena A (2024) Ranking Over Scoring: Towards Reliable and Robust Automated Evaluation of LLM-Generated Medical Explanatory Arguments

42. Kahl K-C, Erkan S, Traub J, Lüth CT, Maier-Hein K, Maier-Hein L, Jaeger PF (2025) SURE-VQA: Systematic Understanding of Robustness Evaluation in Medical VQA Tasks
